# Complete Genome Sequences of Four *Parageobacillus* Strains Isolated from Soil in Japan

**DOI:** 10.1128/mra.00204-22

**Published:** 2022-05-11

**Authors:** Kentaro Miyazaki, Kae Hosoya

**Affiliations:** a Bioproduction Research Institute, National Institute of Advanced Industrial Science and Technology (AIST), Tsukuba, Ibaraki, Japan; b Department of Computational Biology and Medical Sciences, Graduate School of Frontier Sciences, The University of Tokyo, Kashiwa, Chiba, Japan; c International Center for Biotechnology, Osaka University, Suita, Osaka, Japan; d Department of Applied Molecular Chemistry, College of Industrial Technology, Nihon University, Narashino, Chiba, Japan; Indiana University, Bloomington

## Abstract

We isolated four *Parageobacillus* strains from soil in Japan and completely sequenced their genomes. Three of four strains showed ≥98.9% average nucleotide identity (ANI) to Parageobacillus caldoxylosilyticus S1812^T^, while one strain, designated KH3-4, showed the highest ANI (91%) to Parageobacillus thermantarcticus M1^T^, suggesting the species novelty of KH3-4.

## ANNOUNCEMENT

*Parageobacillus* is a genus of betaproteobacteria in the family *Burkholderiaceae* that is Gram-positive and a facultatively anaerobic thermophile. *Parageobacillus* species have great biotechnological potential (1), for example, as a source for thermophilic enzymes (2), fuel production (3, 4), and the bioremediation of environmental pollutants (5). At the time of writing, there are six validly named species in the genus *Parageobacillus* (https://lpsn.dsmz.de/genus/parageobacillus). So far, seven complete genome sequences have been reported for *Parageobacillus*, including for Parageobacillus caldoxylosilyticus (1 strain), Parageobacillus thermoglucosidasius (4 strains), and Parageobacillus toebii (2 strains).

We collected soil samples from the city of Tsukuba, Japan. The samples were suspended in distilled water and spread over Lennox LB agar (1.6% [wt/vol]) plates. After incubation at 65°C overnight, dozens of well-separated single colonies were isolated; colony PCR was conducted to analyze the 16S rRNA genes using a set of primers, Bac8f(C) and UN1542r (6). Among the colonies, four strains, designated KH1-5, KH1-6, KH3-4, and KH3-5, which were expected to belong to the genus *Parageobacillus*, were subjected to complete genome analysis.

To prepare the genomic DNA, cells were grown in 5 mL LB broth at 65°C for 24 h with vigorous shaking (200 rpm). The genomic DNA was purified using a blood and cell culture DNA mini kit (Qiagen). For long-read sequencing, unsheared genomic DNA (1 μg) was treated using a short-read eliminator kit (Circulomics) to remove fragments of <10 Kbp, and a library was constructed using a ligation sequencing kit (Oxford Nanopore Technologies [ONT]). Sequencing was performed using a GridION X5 system on a FLO-MIN106 R9.41 revD flow cell (ONT). Base calling was conducted using Guppy v.4.0.11. The raw sequencing data (Table 1) were filtered (Q < 10; length, <1,000 bases) using NanoFilt v.2.7.1 (7). For short-read sequencing, a library was constructed using an MGIEasy FS PCR free DNA library prep set (MGI) with a ~400 to 500-bp insert. Paired-end sequencing (2 × 150 bases) was then performed on a DNBSEQ-400 instrument (MGI). The raw sequencing data (Table 1) were filtered (Q < 30; length, <20 bases) using fastp v.0.20.1 (8). The trimmed long- and short-read data were assembled using Unicycler v.0.4.8 (9), and the assembly was polished using Pilon v.1.24 (10). Each strain contained a single circular chromosome, and KH3-5 contained one circular plasmid; the circularity was confirmed using Unicycler.

**TABLE 1:** Sequencing metrics for the four *Parageobacillus* strains in this study

Strain	BioSample accession no.	Chromosome or plasmid	DNBSEQ (short-read) data			GridION (long-read) data				Length (bp)	GC content (%)	GenBank accession no.
			No. of paired-end reads	Total length (Mb)	SRA accession no.	No. of reads	*N*_50_ (bp)	Total length (Mb)	SRA accession no.			
*P. caldoxylosilyticus* KH1-5	SAMD00442691	Chromosome	7,592,538	1,139	DRR346603	136,964	5,757	552	DRR346607	3,850,765	44.3	AP025623
*P. caldoxylosilyticus* KH1-6	SAMD00442692	Chromosome	7,161,769	1,074	DRR346604	179,515	9,837	1,244	DRR346608	3,850,773	44.3	AP025624
*Parageobacillus* sp. KH3-4	SAMD00442693	Chromosome	9,226,524	1,384	DRR346605	981,529	4,531	3,090	DRR346609	3,816,932	43.0	AP025627
*P. caldoxylosilyticus* KH3-5	SAMD00442694	Chromosome	7,158,442	1,074	DRR346606	1,107,180	4,644	3,572	DRR346610	3,832,285	44.2	AP025625
		Plasmid (pPcaKH3-5b)								6,889	51.7	AP025626

Automatic annotation was conducted using DFAST v.1.2.15 (11); the genomic features are summarized in Table 1. A JSpecies analysis (12) revealed that KH1-5, KH1-6, and KH3-5 showed ≥98.9% average nucleotide identity (ANI) to each other and to the type strain of *P. caldoxylosilyticus* (strain S1812; GenBank accession number GCF_019272935.1), while KH3-4 showed the highest ANI (91.9%) to the type strain of *P. thermantarcticus* (strain M1; GCF_900111865.1), suggesting the species novelty of KH3-4 (95% ANI being the cutoff for the delineation of a species). For all software, default parameters were used.

### Data availability.

All four *Parageobacillus* strains reported in this paper are associated with BioProject accession number PRJDB12551. The BioSample accession numbers, genome sequences, and raw sequencing data are available under the accession numbers listed in Table 1.
